# Pathological Mineralization: The Potential of Mineralomics

**DOI:** 10.3390/ma12193126

**Published:** 2019-09-25

**Authors:** Elena Tsolaki, Sergio Bertazzo

**Affiliations:** Department of Medical Physics & Biomedical Engineering, University College London, London WC1E 6BT, UK

**Keywords:** mineralomics, calcification, pathological mineralization, ectopic calcification, minerals

## Abstract

Pathological mineralization has been reported countless times in the literature and is a well-known phenomenon in the medical field for its connections to a wide range of diseases, including cancer, cardiovascular, and neurodegenerative diseases. The minerals involved in calcification, however, have not been directly studied as extensively as the organic components of each of the pathologies. These have been studied in isolation and, for most of them, physicochemical properties are hitherto not fully known. In a parallel development, materials science methods such as electron microscopy, spectroscopy, thermal analysis, and others have been used in biology mainly for the study of hard tissues and biomaterials and have only recently been incorporated in the study of other biological systems. This review connects a range of soft tissue diseases, including breast cancer, age-related macular degeneration, aortic valve stenosis, kidney stone diseases, and Fahr’s syndrome, all of which have been associated with mineralization processes. Furthermore, it describes how physicochemical material characterization methods have been used to provide new information on such pathologies. Here, we focus on diseases that are associated with calcium-composed minerals to discuss how understanding the properties of these minerals can provide new insights on their origins, considering that different conditions and biological features are required for each type of mineral to be formed. We show that mineralomics, or the study of the properties and roles of minerals, can provide information which will help to improve prevention methods against pathological mineral build-up, which in the cases of most of the diseases mentioned in this review, will ultimately lead to new prevention or treatment methods for the diseases. Importantly, this review aims to highlight that chemical composition alone cannot fully support conclusions drawn on the nature of these minerals.

## 1. Introduction

Animals [1] and humans [2] all experience biological mineralization processes in different tissues and contexts. Mineralization is a key biological process which, under normal conditions, is responsible for the development of hard tissues, such as bone, cartilage, and teeth, as well as their healing. Here, we discuss and bring attention to a biological mineralization process sometimes overlooked, but with a huge impact on human health: pathological mineralization. 

Such mineralization can take place in practically all soft tissues of humans in connection with the most diverse diseases (Table 1), and consists of different minerals, including calcium phosphates [3,4], calcium carbonates [5], and calcium oxalates [6,7]. Despite the many molecular mechanisms proposed for pathological calcification in most mineralizing diseases, the exact causes and full formation mechanisms of the minerals found in the affected soft tissues are hitherto not completely understood. Research on pathological mineralization has been strongly rooted in the indirect study of the mineral component [8,9,10,11], with a significant number of studies not taking into account the properties of the minerals in vivo, as identified through their direct analysis using material characterization methods including electron microscopy [3] and spectroscopic methods [12].

Physicochemical characterization methods have extensively been used in biological research, leading to a better understanding of organisms and biological processes. These methods include scanning and transmission electron microscopy, which allowed the visualization of infected cells [56,57] and a large number of viruses [56,58], respectively, leading to further important discoveries. Likewise, spectroscopic methods, such as Raman spectroscopy [59], together with X-ray diffraction, have contributed to the discovery of the structure of DNA [60,61,62]. These same methods have also been invaluable in expanding our understanding of biological systems where inorganic components are present [63,64,65]. For example, thermal analysis, infrared spectroscopy and electron microscopy with energy dispersive X-ray spectroscopy have been combined to provide fundamental information on the structure of bone [66,67,68,69,70].

Despite the fact that such a research approach has proven successful countless times, it has surprisingly thus far been restricted to a few specific biological fields (traditionally covering hard tissues, biomaterials, and marine biology systems). Application of these same materials science methods in pathological mineralization could therefore lead to a better understanding of the role, mechanisms, and causes leading to mineral formation in the soft tissue affected by associated diseases. The importance of these minerals and their properties can be seen, for example, in cardiovascular and ocular calcification (which affect large percentages of the general population and are discussed in depth in this review) where mineral formation in itself is largely detrimental to patients.

Recently, the calcification associated with a disease has been increasingly recognized as an important component of that disease, generating a need to understand both organic and inorganic components in order to fully understand a pathology. This research pathway, however, requires multidisciplinary expertise, where the general physicochemical information about the minerals (such as their composition, crystallinity, phase, and morphology) is as important as the information about cells and extracellular matrix. Additionally, in the case of breast cancer, the mineral content of the calcification has only recently become a focus of interest, with results from several studies revealing the importance of further understanding the properties of minerals and how they relate to different breast cancer types.

In this review, by using as examples relevant diseases that present calcification, we argue the case for the direct study of the minerals found in vivo, if we aim to fully understand calcific diseases. Moreover, we point out that the study of several physicochemical properties (such as composition, crystallinity, phase, and morphology) of minerals present in pathologic calcification can provide evidence that distinct mechanisms might potentially be responsible for different minerals being formed in association with different diseases.

For example, distinct mineral phases, including amorphous calcium phosphate and apatite [71,72], can be easily formed in vitro, under specific and controlled conditions, such as at a specific pH [73,74]. However, the formation or transformation of these same minerals in biological systems is more complex. This is due to the presence of different proteins and cells affecting mineralization processes and favouring the formation of specific mineral phases. For instance, in the formation of bone, it is well known that osteoblasts are responsible for bone matrix formation and its regeneration [75,76]. Additionally, some acidic phosphoproteins, such as bone sialoprotein (BSP), have been found to play a major role in the nucleation of bone minerals [77,78]. For instance, the presence of a calcium-containing mineral indicates either that calcium ions are abundant in the extracellular matrix it is formed in or that it arises from cells. These cells could be osteoblasts or, as suggested, macrophages (or even specific cell organelles, such as vesicles and exosomes), which would contain a high amount of calcium. Other than chemical composition and phase, which can hint at an increase in the concentration of specific ions in the extracellular environment, such as in the formation of gallbladder stones [79], characteristics such as crystallinity can hint at the biochemical pathways leading to mineral formation. For example, minerals that diffract as a single crystal, such as those found in shells [80], are usually the product of mechanisms guided by unique proteins that are able to control crystallinity. Indeed, the information about crystallinity provides a hint about the type of proteins involved in the formation of such unique minerals. This is the case even for systems where crystallinity can vary, which include pathological calcification and natural hard tissues, such as bone (whose crystallinity changes over the time) [81,82,83]. In bone and dental minerals, gaining knowledge of changes in the crystallinity of a mineral also gives insights into changes these minerals undergo, ultimately leading to a better understanding of how to prevent such changes [84,85]. Finally, minerals of specific shapes are generally restricted to a certain format by a specific matrix. For example, the architecture of bone is shaped by the presence of collagen fibres restricting mineral deposition to specific spaces [86,87].

In this work, we discuss and emphasize the importance of pathological minerals and review the literature associated with how material science has been applied to achieve a better understanding of pathological mineralization. Additionally, we will discuss in depth some important examples of pathological calcification occurring in the cardiovascular system, breast tissue, kidneys, eyes, brain, and placenta, all of which are associated with major pathologies. The calcification associated with these diseases is highly relevant and affects a large portion of the world population. For example, chronic kidney disease presented 21.3 million new cases in 2016 [88], rheumatic heart diseases presented 33.4 million cases in 2015 [89], breast cancer presented 2.4 million new cases in 2015 [90], age-related macular degeneration presented over 100 million cases in 2014 [91], placental calcification (leading to pregnancy complications) affects most pregnant women [92,93] and, finally, Parkinson’s disease presented around six million new cases in 2016 [94]. Notwithstanding the fact that not all of these pathologies lead directly or indirectly to mineralization processes in every affected individual, their high prevalence means a significant percentage of the world population is facing the adverse, in many cases deadly, effect of soft tissue mineralization.

## 2. Cardiovascular Mineralization

Cardiovascular mineralization is associated with many diseases having high mortality rates, including atherosclerosis [95], aortic valve stenosis [96], chronic kidney disease [97,98], and rheumatic fever [99]. Nevertheless, and despite the large number of proposed mechanisms of mineral formation, the exact processes leading to the minerals observed in vascular tissue are still unsettled, and several competing mechanisms are found in the literature [11,96,100,101,102,103,104,105,106].

It has been previously suggested that the mineralization of cardiac tissues is, in part, a process of bone formation [107], an indication mainly based on the elemental composition of the minerals, on the presence of biological bone markers in the affected tissue and on the transdifferentiation of vascular smooth muscle cells into bone cells. However, X–ray diffraction pattern analysis [108,109], electron microscopy [3,110], and electron diffraction [3] have revealed that the morphology, elemental composition, and crystallinity of cardiovascular mineralization are all remarkably different from bone. In bone, the calcium/phosphorus ratio most commonly reported is below 1.7 [66,111,112,113], whereas the ratio in vascular mineralization (determined by infrared spectroscopy and atomic absorption spectroscopy) has been most frequently reported as 1.7 or above [114,115]. Additionally, and unlike bone, magnesium corresponds to a considerable percentage of the inorganic component of cardiovascular mineralization [114,115,116].

**Figure 1:**
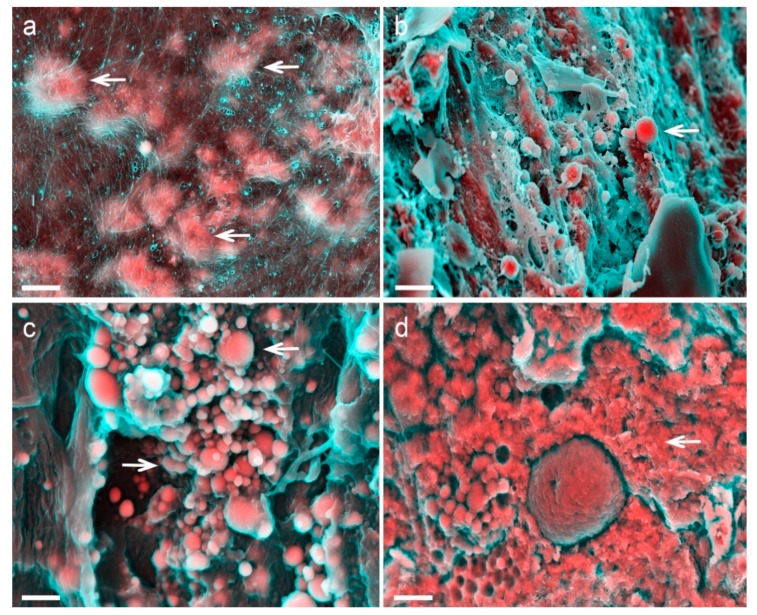
Density-dependent colour-scanning electron micrographs (DDC-SEM) [3] of calcification observed in cardiovascular tissue. Red and pink indicate inorganic and turquoise (blue/green) indicates organic material. (**a**) Calcified fibres indicated by arrows. Scale bar = 2 µm. (**b**) Calcific particles (arrow). Scale bar = 5 µm. (**c**) Aggregates of calcific particles (arrows). Scale bar = 1 µm. (**d**) Large mineral of no defined morphology (arrow). Scale bar = 2 µm.

Also unlike bone, electron microscopy analysis has demonstrated that vascular calcification is formed by three distinct structures: mineralized fibres (Figure 1a), calcific particles (Figure 1b,c), and a large mineral with no defined morphology (Figure 1d). Even more surprising is the distinct crystallinity of these structures. Even though the large mineral presents lower crystallinity, as expected for biological mineralization (but still lower than what is found in bone) [3], electron diffraction analyses showed that the calcific particles diffract as a single crystal [3], which makes them one of the most crystalline minerals found in vertebrates.

Physicochemical characteristics such as crystallinity, chemical composition, and internal structure of the calcified material in cardiovascular tissue potentially hold important information about mineral formation in that tissue. The presence of three distinct materials within that mineralized tissue, for instance, suggests that more than one mechanism of mineralization is taking place. Moreover, the differences among these minerals and between them and the minerals found in bone indicate that the former may have a different mechanism of mineralization than the one found in bone. Ultimately, the differences between the materials present in the cardiovascular tissue and in bone could even suggest that cells other than bone cells are also involved. In summary, these findings highlighted the need for new mineralization pathways being identified [10,11,117,118]. Moreover, they have underlined the need for a range of distinct mechanisms being potentially combined in order to fully understand the origins of cardiovascular calcification (Table 2) and allow further therapeutic advances [119].

## 3. Breast Tissue Mineralization

**Figure 2:**
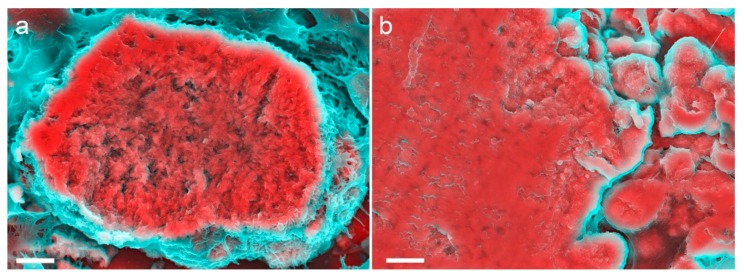
Density-dependent colour-scanning electron micrographs of breast microcalcifications. Red and pink indicate inorganic and turquoise (blue/green) indicates organic material. (**a**) Electron micrograph of a small microcalcification. (**b**) Electron micrograph of a larger microcalcification. Scale bars = 2 µm.

Mineralization of the breast tissue has been suggested to be a result of tissue necrosis as a consequence of injury or a range of diseases, such as chronic kidney disease [120] and hypertension [121]. Additionally, microscopic mineral deposits (known as microcalcifications) (Figure 2) found in breast tissue are a key component in the diagnosis of breast carcinomas [122]. Microcalcifications are also regularly used as mammographic features in the differentiation between malignant and benign diseases [123,124].

This association of breast mineralization to cancer has, therefore, led to a number of research initiatives aiming to identify specific differences between benign and malignant mineralization [125,126,127]. Through electron and light microscopy, X-ray diffraction, and microprobe analysis, two chemically different types of minerals were associated with breast carcinomas: calcium oxalate and apatite [13,14]. Calcium oxalate has been identified mostly in the context of benign diseases [128], although some recent studies failed to identify its presence [129]. On the other hand, apatite has been recorded widely in the literature, in both benign and malignant cases [15,125]. Recent studies, using mainly X-ray microanalysis, have also pointed out the presence of magnesium-substituted apatite in malignant cases [14,16,119,128]. No clear correlation, however, has been found between the levels of magnesium and malignancy [16,129]. Studies where Fourier transform infrared (FTIR) and Raman spectroscopy were used found that a decrease in the concentration of carbonate in the mineral is concomitant to a further malignancy stage [15,125].

Other than their diagnostic capacity, the physicochemical properties of minerals in breast cancer are of growing interest due to their potential to give hints on the prognosis of the disease [130,131]. It has been reported that breast cancer patients with small breast mineralizations have a lower survival rate than patients who do not have such deposits [132], and the presence of minerals in ducts increases the risk of cancer recurrence [131]. Studies have also reported that the presence of apatite in breast cancer cell cultures can enhance mitosis processes [9] and also the migration of tumour cells [133]. The exact origins of these apatite minerals are still unknown, and their characteristics remained unexplored for many years. This is possibly due to the common belief that mineralization had no biological significance and was just only a by-product of cell death (Table 3).

Studies, however, indicate an active cellular process, with in vitro experiments showing that tumourigenic mammary cells are capable of producing apatite, in contrast to non-tumourigenic cells, which do not mineralize [8]. This capability of breast tumour cells to form distinct minerals has been attributed to the fact that such cells express bone-associated proteins [134,135]. It has been therefore suggested that, as a consequence, osteogenic-like processes might be leading to the formation of apatite in the mammary tissue [136].

## 4. Kidney Mineralization

**Figure 3:**
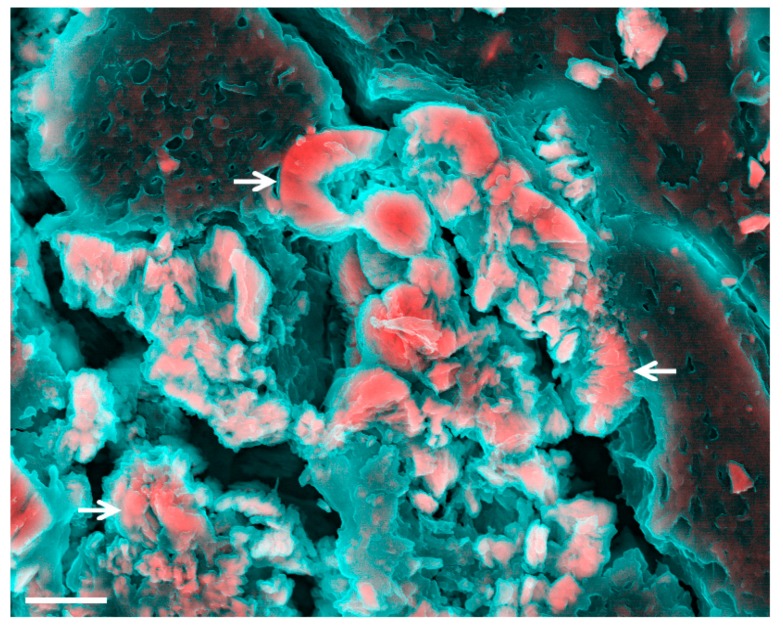
Density-dependent colour-scanning electron micrographs of kidney stones. Red and pink indicate inorganic and turquoise (blue/green) indicates organic material. Electron micrograph of mineral found in the kidney (arrows) [145]. Scale bar = 2 µm.

Kidney stones (Figure 3) are probably the most famous type of pathological mineralization, due to the high prevalence of kidney stone disease (nephrolithiasis) [137]. Millions of people around the world suffer from kidney stone disease, which also carries a high probability of recurrence after treatment [138]. Stones have been observed in patients with systematic diseases, such as hyperparathyroidism [138], and have been associated with obesity [139], diabetes [140], metabolic syndrome [141], and adverse cardiovascular outcomes [142,143]. In the majority of cases, though, they are caused by metabolic derangements leading to urinary imbalances and supersaturation [138,144].

The minerals present in kidney stone disease have been thoroughly studied and a number of different morphologies and chemical compositions have been found [7,146]. Although it is known that renal stones are primarily composed of calcium oxalate, up to 50% of them contain some amount of calcium phosphate [7,40]. Moreover, pure calcium oxalate stones exhibit different chemical phases, such as whewellite and weddellite [41], and a small percentage of stones are also formed by magnesium ammonium phosphate, urate, or cystine [40]. The majority of these are associated with hyperoxaluria, hypercalciuria, as well as low urine pH values [7,146], resulting in mineral formation via supersaturation processes [135]. For example, calcium and oxalate concentrations in urine have a key role in calcium oxalate supersaturation, and similarly, calcium phosphate formation is due to high calcium concentration and pH changes [147].

Due to the heterogeneity of kidney stones, precise information on their elemental composition is critical for the choice of treatment. Such a task would have been impossible without the incorporation of material characterization methods in the diagnostic workflow. Currently, Fourier transform infrared spectroscopy (FTIR), X-ray diffraction, and electron microscopy are used routinely in order to obtain chemical information about the minerals present in kidney stones [41,148,149]. Following diagnosis, the treatment of the disease is then to be chosen from a range of minimally invasive procedures currently used [150] and a wide range of pharmaceutical drugs specific to each stone type [139]. Long-term disease management is also required in order to prevent recurrence, and primarily involves dietary changes [139,151,152,153] since there are no definitive prevention methods in place.

Existing treatment methods focus on the disintegration of already developed stones and can do very little when it comes to their prevention [151], which is usually based on dietary and lifestyle changes designed to tackle the large number of factors resulting in kidney stones that have been identified over the years [151,152]. Despite the great deal of information available on the structure, morphology, and location of the different minerals and the several mineralization models being proposed [154,155], the recurrence of kidney stones is not well understood (Table 4). The reason for this is that the complex interactions between the minerals and the surrounding cells are yet not fully understood [7,144,156,157]. In other words, there is no clear understanding on the short- and long-term effect minerals may have on the biological features around them.

## 5. Ocular Mineralization

Mineralization can also be found in several ocular structures, such as the cornea [158], retina [159], Bruch’s membrane [160], and the optic nerve [161]. Studies indicate that these mineral deposits are usually associated with either trauma or idiopathic causes, affecting most commonly the cornea and retina.

Corneal mineralization is observed in band keratopathy [162,163] and corneal calcareous degeneration [164,165], with both conditions being related to a number of underlying pathologies and inflammation disorders [158]. Interestingly, there is evidence that the mineralization process in the cornea can be also triggered by phosphate buffers, since patients that have undertaken such treatment for healing eye burns have developed corneal mineralization later on [166,167]. Data have also been published supporting a relationship between mineralization and levels of phosphorous in the serum [168]. The chemical composition of this mineral has been identified via energy dispersive X-ray spectroscopy as calcium phosphate [4,160].

Calcium has also been identified in the minerals observed in the optic nerve head (optic disk) [169] and which are referred to as drusen. Optic disk drusen is believed to be caused by defects in the axonal metabolism and blood supply due to a small scleral canal [169,170] and has been associated with Best vitelliform macular dystrophy [171], but not much work has been done in the way of identifying the causes of this type of mineral deposition. More experimental work is also needed to confirm the exact phase of these materials, either in the optic nerve or in the eye as a whole.

An exception to this rule are the minerals present in the retinal pigment epithelium (RPE), which have been studied in much more detail as they have been proven to lead to age-related macular degeneration (AMD) [172], a disease that affects millions of people every year worldwide. In the United Kingdom alone there were 513,000 cases in 2012, with the number estimated to double by 2020 [173,174], making it the leading cause of vision impairment in the elderly population of that country.

In AMD patients, the accumulation of minerals in the RPE appears to block the normal flow of nutrients and waste across the retina, resulting in the degeneration of tissue on either side of the Bruch’s membrane [175]. Interestingly, electron microscopy analysis identified spheres in the mineralized retinal tissue, which were reported to be consisting of calcium phosphate [176]. Furthermore, a study using micro-focused synchrotron X-ray diffraction indicated that part of the mineral is indeed apatite but has also indicated that the spherules are not homogeneous in either structure or composition [160].

**Figure 4:**
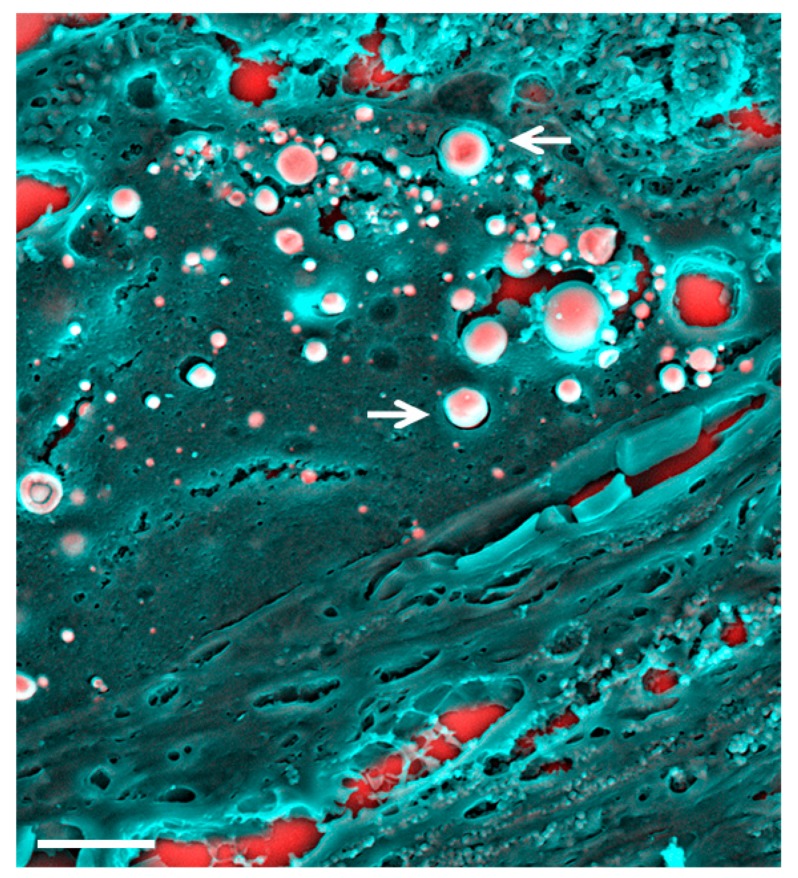
Density-dependent colour-scanning electron micrographs of mineralization observed in the retinal pigment epithelium (RPE). Red and pink indicate inorganic and turquoise (blue/green) indicates organic material. Electron micrograph of spherules (arrows) found in RPE located above Bruch’s membrane. Scale bar = 2 µm.

Recent works have unveiled a total of three distinct structures of minerals present in AMD; spherules (in the RPE), plaques, and nodules (in Bruch’s membrane) [4]. Physicochemical characterization methods have shown that the spherules (Figure 4) consist of whitlockite, the plaques are made of amorphous apatite, and the nodules of apatite [4]. The same study proves that the distinct minerals are associated with different clinical outcomes and suggests that correlation of the outcomes of clinical imaging to the physicochemical properties of the minerals can give insights on the progression of the disease, ultimately contributing to the development of alternative therapeutic methods.

Taken together, the physicochemical characterization methods have unveiled a fair amount of information, which has created possibilities for new targets for the treatment of AMD to be identified. In addition, the identification of distinct minerals in the RPE indicated the presence of different mineralization pathways and lead to the proposal of different formation mechanisms [4,160]; however, the exact origins of these mineral structures and what factors trigger their formation are yet unknown (Table 5).

## 6. Brain Mineralization

**Figure 5:**
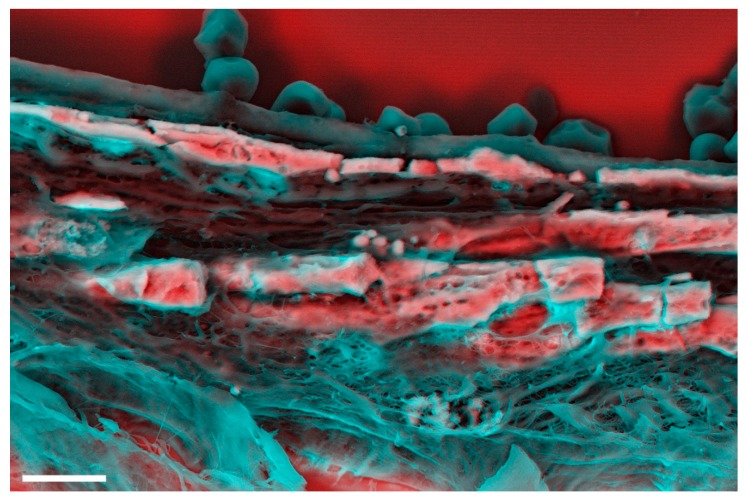
Density-dependent colour-scanning electron micrographs of intracranial vascular calcification. Red and pink indicate inorganic and turquoise (blue/green) indicates organic material. Scale bar = 4 µm.

Mineralization of the brain was first believed to be a result of calcium abnormalities in the body, but it was later suggested that iron and dopamine metabolism abnormalities play a significant role as well [177,178]. As the mechanisms involved in the initial formation of minerals in the brain are not fully understood, the abovementioned abnormalities are often directly related to psychotic pathologies [179]. In the brain, minerals have been reported in, amongst other locations, the basal ganglia, grey matter, and blood vessels. Case studies suggest that the vascular non-atherosclerotic mineralization of structures such as the cerebral cortex, basal ganglia, and cerebellum is related to Fahr’s disease [31]. Additionally, intracranial calcifications are due to meningioma [179], while intracranial arterial calcification (Figure 5) has been proven to increase the probability of ischemic stroke in patients with chronic kidney disease [180].

Although all of these mineralization processes have been associated with calcium phosphate minerals, these are not the only minerals observed in the brain. Histochemical analysis studies on the properties of basal ganglia mineralization have shown that other than calcium, elements such as iron, manganese, zinc, copper, magnesium, and aluminium also accumulate forming minerals [177,181]. In particular, iron minerals have been frequently observed, with studies indicating that the overall amount of iron in the brain increases dramatically with age, concentrating in the grey matter and especially in the globus pallidus, a component of the basal ganglia [182]. Although iron is a fundamental element for the good functioning of the human body in general, it is believed to be potentially responsible for cognitive impairment and has been linked to neurodegenerative disorders such as Parkinson’s disease [183]. However, it is not yet completely clear whether the build-up of iron is a direct cause or simply a collateral effect of those diseases.

One of the limitations in the study of brain mineralization is that most of the studies are carried out with macroscopic methods of mineral characterization (from the microscale up) [184,185,186]. Such macroscopic methods are used to provide information on the location of the mineral in the brain, but no information is collected at the nanoscale, and the physicochemical information recorded is limited. As a result, it is still not clear whether brain mineralization is a passive process, due to ageing, or an active process, nor is it clear whether these minerals have an effect on the progression of associated pathologies (Table 6).

## 7. Placental Mineralization

Up to forty percent of women develop some degree of placental calcification during pregnancy [187]. Of all pregnant women, 18% develop severe mineralization [188], which increases the risk of early preterm labour [189]. While it is associated with pregnancy-induced hypertension (PIH) [190] affecting the mother, the presence of calcium phosphate on placental walls can also have unfavourable effects on the growth and maturation of the foetus and of the placenta itself.

Placental mineralization is divided into different grades, classified according to the Grannum grading system [191]. Mineralization at low amounts has been suggested to be a natural process and harmless through pregnancy, due to the ageing of the placenta and progressive with gestational age [188,192], while extensive mineralization has been shown to carry several risks [193]. The origins of these minerals remain unknown, but a higher incidence has been correlated to smoking [194], passive smoking [195], and bacterial infections [196]. On the other hand, intake of alpha-tocopherol, beta carotene, and vitamin C were found to contribute to the reduction of minerals in placental villi, in some women [194].

As is the case for most other mineralizing pathologies, placental mineralization is formed from apatite [197,198], and little work has been done on the characterization of this mineral. Electron microscopy has revealed that calcifications tend to spread over the whole of the placental surface, where different structures have been observed, including mineralized plaques and large concretions [192], all with a calcium-to-phosphorous ratio lower than what is normally found in bone [198,199]. It has therefore been suggested that placental mineralization is caused by supersaturation of the physiological solutions surrounding the tissue, rather than either the physiological process of bone formation or the dystrophic processes caused by tissue necrosis [198].

Placental mineralization studies have been carried out in a clinical setting [189,193,200] leaving, however, a few open questions (Table 7). Achieving a deeper understanding of the processes of formation and the factors affecting the minerals is thus a fundamental step towards identifying their medical significance.

## 8. Conclusions and Future Perspective

**Figure 6:**
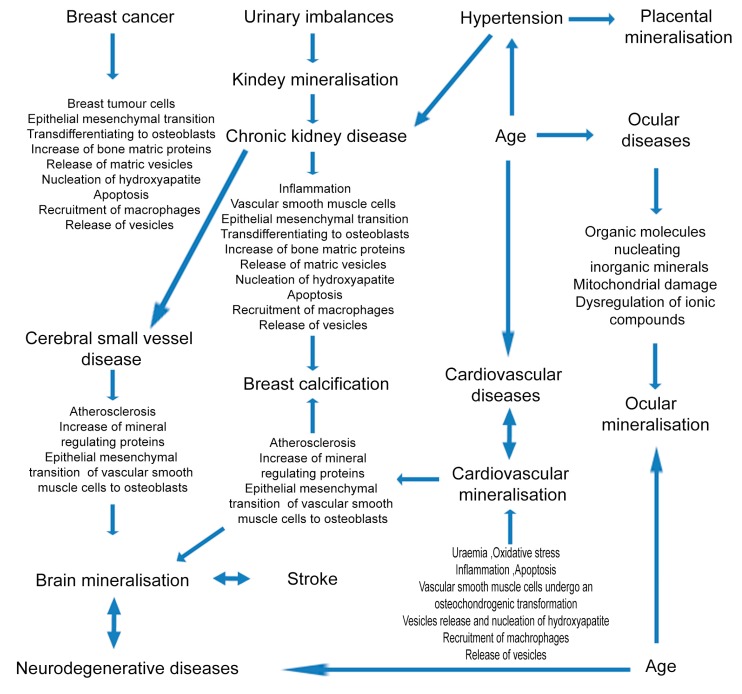
Schematic diagram of a range of pathologies and factors leading to mineralization in different parts of the body, including the proposed mechanisms responsible for mineral formation.

Pathological mineralization in the soft tissues discussed in this review is a result of complex biochemical mechanisms (Figure 6). This review highlights that mineral properties such as crystallinity, chemical phase, elemental composition, appearance, and internal structure of the inorganic components present in calcific diseases have not only added to our understanding of the minerals themselves, but have also generated fundamental knowledge on the development, diagnosis, and treatment of the associated diseases.

The knowledge to be gained from characterizing the chemical composition, crystallinity, and appearance of minerals present in diseases as common as kidney stone disease could drive research and practice to achieve better treatment methods. These could be potentially useful to tackle cardiovascular and ocular diseases, where minerals are a fundamental component of the pathologies themselves. Therefore, preventing the formation or development of minerals can lead to breakthroughs in the treatment of the associated diseases. Breast cancer is another example where further analysis of the minerals will supplement existing knowledge on the relationships between different minerals and different breast cancer types, grades, and stages, ultimately generating data that will lead to more precise breast cancer diagnosis. In the case of other diseases, including brain and placental mineralization, the association, if any, between the inorganic components and the underlying pathologies is not yet clear. In depth analysis of the minerals found in healthy and diseased volunteers will then lead to an understanding of the actual significance of the minerals observed.

Moreover, research strategies focused on the material characterization of minerals should also be applied to in vitro disease models. A comparison between the mineral properties produced in these models and those observed in vivo in healthy and diseased tissues would improve the current understanding of the distinct factors leading to mineral formation in various systems. Some may argue that staining methods, such as alizarin red and Von Kossa staining, commonly used in biomedical research, are invaluable as an initial assessment of in vivo and in vitro samples. A deeper physicochemical characterization of minerals, however, can further provide specific details on their origins and aid the identification of their specific mechanisms of formation. In this way, we may suggest that similar approaches to those used in hard tissue research should be applied to any study where pathological mineralization is present.

Finally, this review stresses that distinct minerals may be found in connection with the same pathology, and these do not necessarily follow the same mechanisms of formation. Once again, the best way to spot such differences is by determining the material characteristics of each distinct mineral, ultimately leading to a better understanding of pathological mineralization and, consequently, of the associated disease.

## Figures and Tables

**Table 1:** List of main diseases associated with pathological mineralization along with information on the affected tissues and chemical composition of the observed minerals. Note: Hydroxyapatite is used in this table for consistency with the literature. We acknowledge, however, that the best word would have been apatite, which can be surrounded by anions other than OH−.

Disease	Mineralization Site	Mineral
Breast cancer	Breast	Hydroxyapatite, calcium oxalate, magnesium-substituted calcium phosphate [13,14,15,16]
Prostate cancer	Prostate	Calcium carbonate phosphate, hydroxyapatite, calcium oxalate monohydrate, calcium oxalate dehydrate, whitlockite [17,18,19,20]
Chronic kidney disease	Vascular tissue	Hydroxyapatite, calcium phosphate [21,22]
Benign prostatic hyperplasia	Prostate	Hydroxyapatite, calcium oxalate monohydrate, calcium oxalate dehydrate [23,24]
Pancreatic cancer	Pancreas	Calcite [25,26]
Ovarian cancer	Ovaries	Calcium phosphate [27,28]
Thyroid cancer	Thyroid	Carbonated calcium phosphate, hydroxyapatite, amorphous carbonated, calcium phosphate apatite, octacalcium phosphate pentahydrate, brushite, whewellite, weddellite, caoxite [29,30]
Fahr’s syndrome	Basal ganglia	Calcium phosphate, calcium carbonate [31,32]
Systemic sclerosis (scleroderma)	Connective tissue	Hydroxyapatite [33,34]
Calcific tendonitis	Tendons	Calcium carbonate apatite, hydroxyapatite [35,36,37,38,39]
Kidney stones (renal calculi)	Kidneys	Magnesium ammonium phosphate, hydroxyapatite, whewellite, weddellite, struvite, urate, cystine [7,40,41,42]
Urinary stasis	Bladder	Whewellite, struvite, ammonium urate, cystine, carbapatite [43,44,45]
Hypoparathyroidism	Basal ganglia	Calcium phosphate [46,47]
Atherosclerosis	Cardiovascular tissue	Hydroxyapatite, whitlockite [48,49]
Calcific aortic valve disease	Aortic valve	Hydroxyapatite [3]
Age-related macular degeneration	Eyes	Apatite, whitlockite [4]
Alzheimer’s disease	Brain	Iron oxide, calcium salts [50,51]
Tuberculosis	Lungs	Calcium phosphate [52]
Meningioma	Brain	Calcium salts [53]
Salivary stones	Saliva glands	Carbonated apatite, whewellite, weddellite, brushite, struvite [54]
Pulp stones	Dental pulp	Calcium phosphate [55]

**Table 2:** List of main questions that remain unanswered in relation to cardiovascular calcification.

Main Questions Remaining Unanswered
• What is the role of the distinct mineral structures in cardiovascular diseases? • Which of the proposed mechanisms of formation are responsible for each mineral structure? • How can cardiovascular calcification be prevented and treated?

**Table 3:** List of main questions that remain unanswered in relation to breast calcification.

Main Questions Remaining Unanswered
• What is the role of the distinct mineral structures in breast cancer and other diseases? • Why do different types of disease lead to the formation of different minerals? • Are these minerals involved in any way in the progression of breast cancer? • Does the presence of the minerals in any way affect the progression of breast cancer? • What are the exact cellular origins of the different minerals observed?

**Table 4:** List of main questions that remain unanswered in relation to kidney mineralization.

Main Questions Remaining Unanswered
• What is the role of the distinct mineral structures in the associated diseases? • What effect do the minerals have on the surrounding tissue? • What causes the recurrence of kidney stones? • How can the formation or recurrence of kidney stones be prevented?

**Table 5:** List of main questions that remain unanswered in relation to ocular calcification.

Main Questions Remaining Unanswered
• What are the exact mechanisms of formation of each of the structures observed? • How can ocular mineralization in general be prevented? • Can AMD be treated completely through the targeting of minerals present in the disease? • Can we prevent the formation of minerals in the tissue?

**Table 6:** List of main questions that remain unanswered in relation to brain calcification.

Main Questions Remaining Unanswered
• What are the physicochemical properties of the minerals observed? • What is the exact microscopic location of the minerals? • How are any of the mineral structures related to any of the diseases they have been associated with? • Do the minerals have any role in the progression of the diseases? • Is the mineral deposition only a passive process resulting from ageing?

**Table 7:** List of main questions that remain unanswered in relation to placental calcification.

Main Questions Remaining Unanswered
• What is the clinical significance of placental mineralization (if any)? • How are these minerals produced? • What are the physicochemical properties of the minerals observed? • Can placental mineralization be prevented or treated?
